# 2,3,6,7-Tetra­meth­oxy-9,10-anthra­quinone

**DOI:** 10.1107/S1600536812033119

**Published:** 2012-07-28

**Authors:** Akira Ohta, Kazuki Hattori, Takeshi Kawase, Takashi Kobayashi, Hiroyoshi Naito, Chitoshi Kitamura

**Affiliations:** a Department of Chemistry, Faculty of Science, Shinshu University, 3-1-1 Asahi, Matsumoto, Nagano 390-8621, Japan; b Department of Materials Science and Chemistry, Graduate School of Engineering, University of Hyogo, 2167 Shosha, Himeji, Hyogo 671-2280, Japan; c Department of Physics and Electronics, Graduate School of Engineering, Osaka Prefecture University, 1-1 Gakuencho, Naka-ku, Sakai, Osaka 599-8531, Japan

## Abstract

Mol­ecules of the title compound, C_18_H_16_O_6_, are almost planar [maximum deviation = 0.096 (4) Å] and reside on crystallographic centres of inversion. They adopt a conformation in which the C_meth­yl_—O bonds are directed along the mol­ecular short axis [C—C—O—C torsion angles of −175.3 (3) and 178.2 (3)°]. In the crystal, mol­ecules adopt a slipped-parallel arrangement with π–π stacking inter­actions along the *a* axis with an inter­planar distance of 3.392 (4) Å. Weak C—H⋯O inter­actions link the mol­ecules into sheets parallel to (10-2).

## Related literature

For a study of the effects of alk­oxy substituents on the structures and solid-state photophysics, see: Ohta *et al.* (2012 ▶). For the synthesis, see: Boldt (1967 ▶). For a related structure, see: Kitamura *et al.* (2009 ▶).
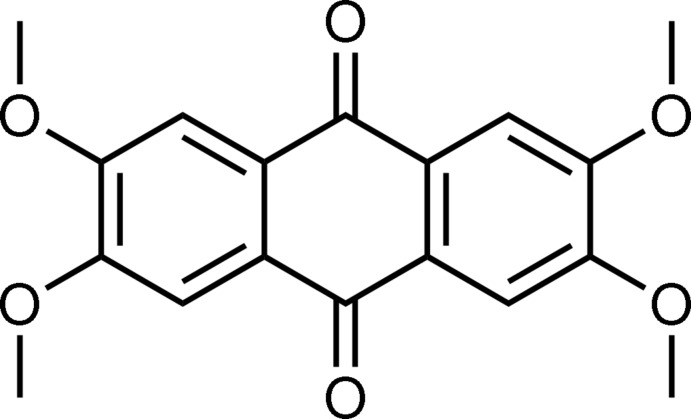


## Experimental

### 

#### Crystal data

C_18_H_16_O_6_
*M* *_r_* = 328.31
Triclinic, 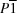

*a* = 4.6607 (4) Å
*b* = 8.4769 (9) Å
*c* = 9.8110 (9) Å
α = 94.859 (3)°
β = 91.410 (2)°
γ = 97.278 (2)°
*V* = 382.87 (6) Å^3^
*Z* = 1
Mo *K*α radiation
μ = 0.11 mm^−1^
*T* = 223 K
0.50 × 0.06 × 0.05 mm

#### Data collection

Rigaku R-AXIS RAPID diffractometer
3738 measured reflections
1725 independent reflections
977 reflections with *I* > 2σ(*I*)
*R*_int_ = 0.027

#### Refinement

*R*[*F* ^2^ > 2σ(*F* ^2^)] = 0.054
*wR*(*F* ^2^) = 0.234
*S* = 1.16
1725 reflections
111 parameters
H-atom parameters constrained
Δρ_max_ = 0.39 e Å^−3^
Δρ_min_ = −0.44 e Å^−3^

### 

Data collection: *RAPID-AUTO* (Rigaku, 1999 ▶); cell refinement: *PROCESS-AUTO*; data reduction: *PROCESS-AUTO*; program(s) used to solve structure: *SIR2004* (Burla *et al.*, 2005 ▶); program(s) used to refine structure: *SHELXL97* (Sheldrick, 2008 ▶); molecular graphics: *ORTEP-3 for Windows* (Farrugia, 1997 ▶); software used to prepare material for publication: *WinGX* (Farrugia, 1999 ▶).

## Supplementary Material

Crystal structure: contains datablock(s) global, I. DOI: 10.1107/S1600536812033119/gg2091sup1.cif

Structure factors: contains datablock(s) I. DOI: 10.1107/S1600536812033119/gg2091Isup2.hkl

Supplementary material file. DOI: 10.1107/S1600536812033119/gg2091Isup3.cml

Additional supplementary materials:  crystallographic information; 3D view; checkCIF report

## Figures and Tables

**Table 1:** Hydrogen-bond geometry (Å, °)

*D*—H⋯*A*	*D*—H	H⋯*A*	*D*⋯*A*	*D*—H⋯*A*
C8—H8*A*⋯O1^i^	0.97	2.58	3.391 (5)	142
C9—H9*B*⋯O2^ii^	0.97	2.54	3.494 (4)	168

Symmetry codes: (i) 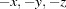; (ii) 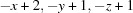.

**Fig. 1.**
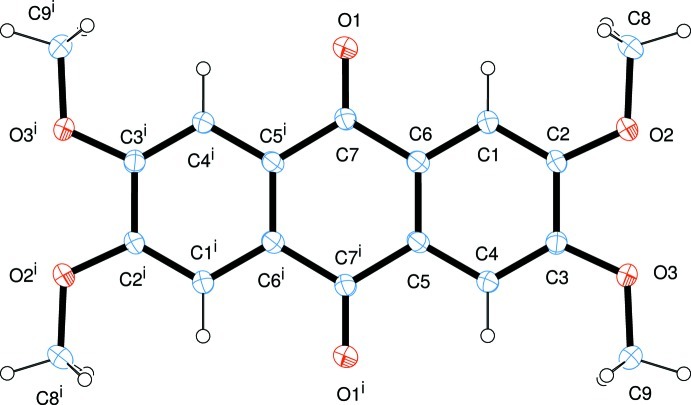
The molecular structure of the title compound (I), showing the atomic numbering numbering and 30% probability displacement ellipsoids. Symmetry code: (i) -x, -y + 1, -z.

**Fig. 2.**
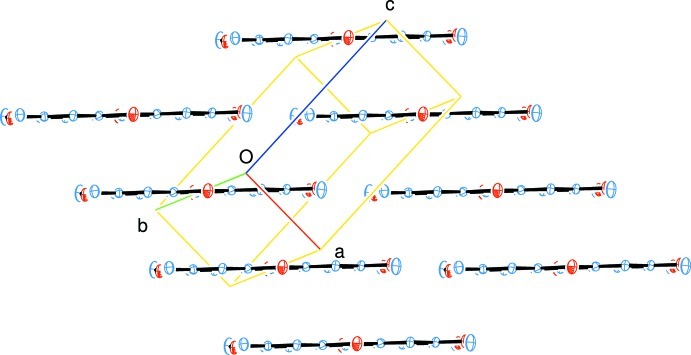
The packing diagram of (I). Hydrogen atoms are omitted for clarity.

## supplementary crystallographic information

### Comment

9,10-Anthraquinone is an important molecule in the field of industrial dyes. We have recently been interested in the tuning of the solid-state optical properties by the introduction of substituents. As part of our program aimed at the elucidation of the effects of alkoxy substituents on the optical properties in the solid state (Ohta, *et al.*, 2012), we are in need of the information on the crystal structures of a variety of 2,3,6,7-tetraalkoxy-9,10-anthraquinones in order to clarify the correlation between crystal structures and the solid-state photophysics. Although the title compound is already known (Boldt, 1967), the X-ray structure was not reported to date. We report herein the crystal structure of the title compound (I).

The molecular structure of the title compound (I) is shown in Fig. 1. The molecule possesses a center of inversion, and half of the formula unit is crystallographically independent. The molecule is almost planar with the maximum deviation of 0.096 (4) Å for C8. The displacements of atoms O1, O2, O3, C8 and C9 relative to the plane of the anthraquinone framework are 0.023 (2), -0.002 (2), 0.013 (2), 0.096 (4), and 0.060 (3), respectively. The molecule prefers the conformation in which the C_methyl_—O bonds are directed along the molecular short axis. The torsion angles of C3—C2—O2—C8 and C2—C3—O3—C9 are -175.3 (3) and 178.2 (3)°, respectively. This conformation is similar to the corresponding moiety in 2,3-dimethoxy-5,12-tetracenequinone (Kitamura, *et al.*, 2009). The molecules adopt a slipped-parallel arrangement as shown in Fig. 2. Then molecules are π-stacked along the *a* axis with an interplanar distance 3.392 (4) Å.

To examine the influence of crystal packing on the solid-state fluorescences, the fluorescence spectrum and the absolute quantum yield of (I) were measured with a Hamamatsu Photonics PMA11 calibrated optical multichannel analyzer with a solid-state blue laser (*λ*_ex_ = 377 nm) and a Labsphere IS IS-040-SF integrating sphere, respectively. The crystals showed negligible fluorescence (*Φ* = 0.002). The fluorescence quenching would be due to the π-stacked structure.

### Experimental

The title compound was prepared according to the literature procedure (Boldt, 1967). Single crystals suitable for X-ray analysis were obtained by recrystallization from DMF.

### Refinement

All the H atoms were positioned geometrically and refined using a riding model with C—H = 0.94 Å and *U*_iso_(H) = 1.2*U*_eq_(C) for aromatic C—H, and C—H = 0.97 Å and *U*_iso_(H) = 1.5*U*_eq_(C) for CH_3_. The positions of methyl H atoms were optimized rotationally.

### Crystal data

**Table:**

C_18_H_16_O_6_	*Z* = 1
*M**_r_* = 328.31	*F*(000) = 172
Triclinic, *P*1	*D*_x_ = 1.424 Mg m^−^^3^
Hall symbol: -P 1	Mo *K*α radiation, λ = 0.71073 Å
*a* = 4.6607 (4) Å	Cell parameters from 1977 reflections
*b* = 8.4769 (9) Å	θ = 3.1–27.5°
*c* = 9.8110 (9) Å	µ = 0.11 mm^−^^1^
α = 94.859 (3)°	*T* = 223 K
β = 91.410 (2)°	Needle, yellow
γ = 97.278 (2)°	0.50 × 0.06 × 0.05 mm
*V* = 382.87 (6) Å^3^	

### Data collection

**Table:**

Rigaku R-AXIS RAPID diffractometer	977 reflections with *I* > 2σ(*I*)
Radiation source: fine-focus sealed x-ray tube	*R*_int_ = 0.027
Graphite monochromator	θ_max_ = 27.5°, θ_min_ = 3.1°
Detector resolution: 10 pixels mm^-1^	*h* = −6→5
ω scans	*k* = −10→10
3738 measured reflections	*l* = −12→12
1725 independent reflections	

### Refinement

**Table:**

Refinement on *F*^2^	Primary atom site location: structure-invariant direct methods
Least-squares matrix: full	Secondary atom site location: difference Fourier map
*R*[*F*^2^ > 2σ(*F*^2^)] = 0.054	Hydrogen site location: inferred from neighbouring sites
*wR*(*F*^2^) = 0.234	H-atom parameters constrained
*S* = 1.16	*w* = 1/[σ^2^(*F*_o_^2^) + (0.1038*P*)^2^ + 0.301*P*] where *P* = (*F*_o_^2^ + 2*F*_c_^2^)/3
1725 reflections	(Δ/σ)_max_ < 0.001
111 parameters	Δρ_max_ = 0.39 e Å^−^^3^
0 restraints	Δρ_min_ = −0.44 e Å^−^^3^

### Special details

**Table:**

Geometry. All s.u.'s (except the s.u. in the dihedral angle between two l.s. planes) are estimated using the full covariance matrix. The cell s.u.'s are taken into account individually in the estimation of s.u.'s in distances, angles and torsion angles; correlations between s.u.'s in cell parameters are only used when they are defined by crystal symmetry. An approximate (isotropic) treatment of cell s.u.'s is used for estimating s.u.'s involving l.s. planes.
Refinement. Refinement of *F*^2^ against ALL reflections. The weighted *R*-factor *wR* and goodness of fit *S* are based on *F*^2^, conventional *R*-factors *R* are based on *F*, with *F* set to zero for negative *F*^2^. The threshold expression of *F*^2^ > 2σ(*F*^2^) is used only for calculating *R*-factors(gt) *etc*. and is not relevant to the choice of reflections for refinement. *R*-factors based on *F*^2^ are statistically about twice as large as those based on *F*, and *R*- factors based on ALL data will be even larger.

### Fractional atomic coordinates and isotropic or equivalent isotropic displacement parameters (Å^2^)

**Table:**

	*x*	*y*	*z*	*U*_iso_*/*U*_eq_	
C1	0.3162 (6)	0.2759 (3)	0.1180 (3)	0.0304 (7)	
H1	0.2882	0.1685	0.082	0.036*	
C2	0.5104 (6)	0.3236 (3)	0.2269 (3)	0.0282 (7)	
C3	0.5524 (6)	0.4852 (3)	0.2811 (3)	0.0279 (6)	
C4	0.3981 (6)	0.5939 (3)	0.2252 (3)	0.0293 (7)	
H4	0.425	0.7012	0.2616	0.035*	
C5	0.2021 (6)	0.5460 (3)	0.1150 (3)	0.0264 (6)	
C6	0.1606 (6)	0.3872 (3)	0.0611 (3)	0.0274 (6)	
C7	−0.0418 (6)	0.3343 (3)	−0.0564 (3)	0.0286 (6)	
O1	−0.0753 (5)	0.1938 (3)	−0.1056 (2)	0.0421 (6)	
C8	0.6538 (9)	0.0647 (4)	0.2327 (4)	0.0529 (10)	
H8A	0.4579	0.0121	0.2389	0.079*	
H8B	0.7865	0.0095	0.2828	0.079*	
H8C	0.7052	0.0628	0.1374	0.079*	
O2	0.6714 (5)	0.2264 (2)	0.2899 (2)	0.0369 (6)	
C9	0.8062 (7)	0.6813 (4)	0.4442 (3)	0.0382 (8)	
H9A	0.8839	0.7476	0.3745	0.057*	
H9B	0.946	0.6892	0.5202	0.057*	
H9C	0.6283	0.7173	0.4764	0.057*	
O3	0.7479 (5)	0.5185 (2)	0.3876 (2)	0.0345 (6)	

### Atomic displacement parameters (Å^2^)

**Table:**

	*U*^11^	*U*^22^	*U*^33^	*U*^12^	*U*^13^	*U*^23^
C1	0.0295 (15)	0.0273 (15)	0.0345 (15)	0.0050 (11)	−0.0052 (12)	0.0030 (12)
C2	0.0270 (14)	0.0262 (14)	0.0325 (14)	0.0079 (11)	−0.0031 (12)	0.0033 (11)
C3	0.0277 (14)	0.0278 (15)	0.0280 (14)	0.0026 (11)	−0.0024 (11)	0.0040 (11)
C4	0.0292 (14)	0.0235 (14)	0.0348 (15)	0.0034 (11)	−0.0048 (12)	0.0022 (11)
C5	0.0254 (14)	0.0226 (14)	0.0320 (14)	0.0048 (11)	0.0015 (12)	0.0039 (11)
C6	0.0274 (14)	0.0254 (15)	0.0299 (14)	0.0045 (11)	0.0015 (12)	0.0037 (11)
C7	0.0262 (14)	0.0255 (14)	0.0342 (15)	0.0040 (11)	−0.0034 (12)	0.0030 (11)
O1	0.0474 (14)	0.0269 (12)	0.0514 (14)	0.0090 (9)	−0.0156 (11)	−0.0022 (9)
C8	0.062 (2)	0.0276 (17)	0.070 (2)	0.0148 (15)	−0.025 (2)	−0.0001 (16)
O2	0.0405 (12)	0.0287 (12)	0.0423 (12)	0.0116 (9)	−0.0126 (10)	0.0013 (9)
C9	0.0418 (18)	0.0317 (17)	0.0392 (16)	0.0025 (13)	−0.0133 (14)	−0.0007 (13)
O3	0.0377 (12)	0.0289 (11)	0.0356 (11)	0.0049 (8)	−0.0145 (9)	−0.0002 (8)

### Geometric parameters (Å, º)

**Table:**

C1—C2	1.382 (4)	C6—C7	1.474 (4)
C1—C6	1.404 (4)	C7—O1	1.236 (3)
C1—H1	0.94	C7—C5^i^	1.479 (4)
C2—O2	1.359 (3)	C8—O2	1.428 (4)
C2—C3	1.414 (4)	C8—H8A	0.97
C3—O3	1.356 (3)	C8—H8B	0.97
C3—C4	1.379 (4)	C8—H8C	0.97
C4—C5	1.397 (4)	C9—O3	1.434 (3)
C4—H4	0.94	C9—H9A	0.97
C5—C6	1.392 (4)	C9—H9B	0.97
C5—C7^i^	1.479 (4)	C9—H9C	0.97
			
C2—C1—C6	120.2 (3)	O1—C7—C6	121.0 (2)
C2—C1—H1	119.9	O1—C7—C5^i^	120.7 (2)
C6—C1—H1	119.9	C6—C7—C5^i^	118.3 (2)
O2—C2—C1	125.2 (3)	O2—C8—H8A	109.5
O2—C2—C3	114.9 (2)	O2—C8—H8B	109.5
C1—C2—C3	119.9 (2)	H8A—C8—H8B	109.5
O3—C3—C4	125.5 (3)	O2—C8—H8C	109.5
O3—C3—C2	114.8 (2)	H8A—C8—H8C	109.5
C4—C3—C2	119.7 (2)	H8B—C8—H8C	109.5
C3—C4—C5	120.5 (3)	C2—O2—C8	117.2 (2)
C3—C4—H4	119.7	O3—C9—H9A	109.5
C5—C4—H4	119.7	O3—C9—H9B	109.5
C6—C5—C4	119.9 (3)	H9A—C9—H9B	109.5
C6—C5—C7^i^	120.8 (2)	O3—C9—H9C	109.5
C4—C5—C7^i^	119.3 (2)	H9A—C9—H9C	109.5
C5—C6—C1	119.7 (3)	H9B—C9—H9C	109.5
C5—C6—C7	120.9 (2)	C3—O3—C9	117.4 (2)
C1—C6—C7	119.3 (3)		
			
C6—C1—C2—O2	179.8 (3)	C4—C5—C6—C7	−179.1 (3)
C6—C1—C2—C3	0.0 (4)	C7^i^—C5—C6—C7	0.6 (5)
O2—C2—C3—O3	0.2 (4)	C2—C1—C6—C5	−0.1 (4)
C1—C2—C3—O3	−180.0 (3)	C2—C1—C6—C7	179.0 (3)
O2—C2—C3—C4	−179.5 (3)	C5—C6—C7—O1	178.8 (3)
C1—C2—C3—C4	0.3 (4)	C1—C6—C7—O1	−0.3 (4)
O3—C3—C4—C5	179.9 (3)	C5—C6—C7—C5^i^	−0.6 (5)
C2—C3—C4—C5	−0.4 (4)	C1—C6—C7—C5^i^	−179.7 (3)
C3—C4—C5—C6	0.3 (4)	C1—C2—O2—C8	4.9 (5)
C3—C4—C5—C7^i^	−179.4 (3)	C3—C2—O2—C8	−175.3 (3)
C4—C5—C6—C1	0.0 (4)	C4—C3—O3—C9	−2.1 (4)
C7^i^—C5—C6—C1	179.7 (3)	C2—C3—O3—C9	178.2 (3)

Symmetry code: (i) −*x*, −*y*+1, −*z*.

### Hydrogen-bond geometry (Å, º)

**Table:**

*D*—H···*A*	*D*—H	H···*A*	*D*···*A*	*D*—H···*A*
C8—H8*A*···O1^ii^	0.97	2.58	3.391 (5)	142
C9—H9*B*···O2^iii^	0.97	2.54	3.494 (4)	168

Symmetry codes: (ii) −*x*, −*y*, −*z*; (iii) −*x*+2, −*y*+1, −*z*+1.
